# Quantitative kinetic modelling and mapping of cerebral glucose transport and metabolism using glucoCESL MRI

**DOI:** 10.1177/0271678X221108841

**Published:** 2022-06-23

**Authors:** Ben R Dickie, Tao Jin, Ping Wang, Rainer Hinz, William Harris, Hervé Boutin, Geoff JM Parker, Laura M Parkes, Julian C Matthews

**Affiliations:** 1 Division of Neuroscience and Experimental Psychology, Faculty of Biology, Medicine and Health, The University of Manchester, Manchester, UK; 2 Geoffrey Jefferson Brain Research Centre, Manchester Academic Health Science Centre, Manchester, UK; 3 Department of Radiology, University of Pittsburgh, Pittsburgh, Pennsylvania, USA; 4 Division of Informatics, Imaging, and Data Science, Faculty of Biology Medicine and Health, University of Manchester, Manchester, UK; 5 Bioxydyn Limited, Manchester, UK; 6 Centre for Medical Image Computing, Department of Medical Physics & Biomedical Engineering and Department of Neuroinflammation, University College London, London, UK

**Keywords:** GlucoCESL, glucoCEST, cerebral glucose metabolism, kinetic modelling, brain tumours

## Abstract

Chemical-exchange spin-lock (CESL) MRI can map regional uptake and utilisation of glucose in the brain at high spatial resolution (i.e sub 0.2 mm^3^ voxels). We propose two quantitative kinetic models to describe glucose-induced changes in tissue *R*_1ρ_ and apply them to glucoCESL MRI data acquired in tumour-bearing and healthy rats. When assuming glucose transport is saturable, the maximal transport capacity (*T*_max_) measured in normal tissue was 3.2 ± 0.6 µmol/min/mL, the half saturation constant (*K*_t_) was 8.8 ± 2.2 mM, the metabolic rate of glucose consumption (*MR*_glc_) was 0.21 ± 0.13 µmol/min/mL, and the cerebral blood volume (*v*_b_) was 0.006 ± 0.005 mL/mL. Values in tumour were: *T*_max_ = 7.1 ± 2.7 µmol/min/mL, *K*_t_ = 14 ± 1.7 mM, *MR*_glc_ = 0.22 ± 0.09 µmol/min/mL, *v*_b_ = 0.030 ± 0.035 mL/mL. *T*_max_ and *K*_t_ were significantly higher in tumour tissue than normal tissue (p = 0.006 and p = 0.011, respectively). When assuming glucose uptake also occurs via free diffusion, the free diffusion rate (*k*_d_) was 0.061 ± 0.017 mL/min/mL in normal tissue and 0.12 ± 0.042 mL/min/mL in tumour. These parameter estimates agree well with literature values obtained using other approaches (e.g. NMR spectroscopy).

Cerebral glucose metabolism is altered in a range of neurological diseases including brain tumours,^1^ Alzheimer's disease,^2^ Parkinson's disease,^3,4^ and multiple sclerosis.^5,6^ The metabolic rate of glucose consumption (*MR*_glc_) is dependent on the rate of glucose transport across the blood-brain barrier (BBB) and downstream utilisation by neurons and astrocytes. When the BBB is intact, glucose enters the brain via the GLUT1 glucose transporter located on endothelial cell membranes, and moves into cells primarily via GLUT1 on astrocyte cell membranes and GLUT3 on neuronal cell membranes.^7^ Movement of glucose through glucose transporters can be approximated by Michaelis-Menten kinetics, with the rate being saturable and dependent on the half saturation constant (also called the binding constant), *K*_t_, and the maximum transport capacity, *T*_max._^8^ Different glucose transporters have different *K*_t_ values, and thus *in-vivo* measurement of *K*_t_ may provide insight into their relative distribution on cellular membranes and alterations to this distribution caused by disease. The maximum transport capacity depends on the availability of transporters (i.e. their density), and similarly in-vivo measurements of *T*_max_ could provide important information regarding the effects of ageing or pathology on glucose delivery to the brain.

In some solid tumours, glucose transporter expression is increased,^9^ which has been shown to correlate with tumour hypoxia, a property associated with treatment resistance and tumour agressiveness.^10,11^ Due to aberrant vessel formation during neo-angiogenesis, BBB integrity may also be compromised, leading to increased free diffusion of glucose across the blood-tumour interface. In neurodegenerative diseases such as Alzheimer's disease, glucose transporter expression is reduced^7,12^ which may starve the brain of glucose,^13^ and contribute towards neurodegeneration.^14^ In many diseases, alterations to glucose transport and/or metabolism are regionally specific, and thus being able to detect glucose transport and utilisation at high spatial resolution across multiple brain regions, would greatly progress our ability to study deficiencies in glucose transport and metabolic dysfunction.

2-deoxy-2-[^18^F]fluoro-D-glucose positron emission tomography (FDG-PET) is the gold-standard technique for imaging glucose transport and metabolism *in-vivo,* and has led to improved understanding of metabolic dysfunction across a range of brain diseases.^15,16^ Typically, trace amounts of FDG are injected and late static tissue PET measurements made. For full kinetic modelling, and in order to derive rates of cerebral glucose transport and utilisation, FDG kinetics in arterial blood and tissue are needed from arterial blood sampling and dynamic PET scanner measurements.^17,18^ FDG differs in its transport and phosphorylation properties relative to glucose and the estimated rate constants derived using FDG must be converted to glucose rate constants using an experimentally derived lumped constant.^19,20^ Several attempts have been made to measure the lumped constant for FDG, with values ranging from 0.71–0.89^21,22^ in rat and 0.68–0.89^20,23–25^ in human, presenting a significant potential source of bias. Quantification of glucose metabolic rates in brain tumours using FDG is further complicated by the unknown redistribution of glucose transporters.

Detection of glucose *in-vivo* in the brain is possible using magnetic resonance spectroscopy (MRS), as first reported by Frahm et al. in 1991.^3,26^ ^1^H MRS has been used to measure uptake and utilisation of glucose following hyperglycaemic challenge, measuring changes in the glucose signals at 3.44 and 5.23 ppm.^27–29^ However, quantification is difficult due to spectral overlap with more concentrated metabolites such as *myo*-inositol, creatine/phosphocreatine and amino acids. Significant effort has been invested in developing methods to improve the specificity to glucose, and downstream metabolites using ^13^C-labelling,^27,30–36^ and more recently, ^2^H-labelled glucose.^37–39^ In both cases, the label passes down the glycolytic pathway, and can be detected in glucose itself and downstream metabolites such as lactate and glutamate. A key advantage of these approaches is reduced spectral overlap, because unlabelled endogenous molecules do not contribute to the spectra. However, these methods are still limited by low sensitivity, meaning large voxel volumes are required to provide adequate signal to noise for precise quantification. Usually, specialised coils^36^ are required for ^13^C and ^2^H approaches, limiting translation, though there are approaches to detect the presence of ^13^C-labelled metabolites in ^2^HMRS that do not require either a ^13^C-channel or ^13^C –tuned RF coil.^40^ Nevertheless, this latter approach lacks the sensitivity to detect glucose itself, being limited to detection of the higher concentration downstream metabolite, glutamate.

More recently, chemical exchange saturation transfer (CEST) and chemical-exchange spin lock (CESL) MRI have been used to indirectly detect glucose via chemical exchange of protons in the hydroxyl groups (^−^OH) of glucose with protons in water. These approaches are derived from techniques that image exchangeable protons in macromolecules^41,42^ and more recently have been used to spatially map glucose uptake in living tissues, and appear to overcome the low detection sensitivity limitations of spectroscopic approaches. CEST was first applied to map uptake of intravenously administered glucose in tumours by Chan et al.^43^ then Walker-Samuel et al..^44^ Since then, CEST and CESL have been applied to detect glucose uptake in rodent and human brain by a number of groups.^45–53^ Jin et al.^46^ and others^50,54–56^ have performed key studies in normal brain, tumour, and stroke showing that glucoCESL and glucoCEST uptake curves are sensitive to both uptake and metabolism of glucose.^50,54^ In these studies, the uptake curves of D-glucose and/or glucose-analog 3-O-methyl-D-glucose (3OMG) were monitored using CESL or CEST MRI. D-glucose and 3OMG are taken up by tissues at similar rates, but 3OMG, which is not metabolised, was found to remain at high levels, while the curves for D-glucose decreased to pre-injection levels. Despite these studies showing the clear sensitivity of CESL and CEST to the transport *and* metabolism of glucose, and the potential for mapping these processes at high spatial resolution, kinetic modelling to determine physiological rate constants has not been applied to this type of data.

In this study, we introduce the theoretical background for quantitative kinetic modelling of glucoCESL MRI data. We evaluate the sensitivity of *R*_1ρ_ to all kinetic model parameters, evaluate bias introduced due to inaccurate glucose input functions, and apply the models to data acquired in tumour-bearing (9 L glioma) and healthy rats. We evaluate two kinetic models describing purely saturable transport (model 1) and mixed transport with saturable and free diffusion components (model 2). The fit quality of each model is compared using the Akaike information criterion on a voxel wise level. Finally, we evaluate differences in the central tendency and distribution (standard deviation) of estimated transport and metabolic parameters between tumour and normal tissue.

## Relaxation model

In the presence of an on-resonant spin-lock pulse, the spin-lock relaxation rate $R_{1\rho }$ can be written in terms of chemical-exchange and non-chemical exchange contributions:^46^

(1) $$R_{1\rho }=R_{ex}+R_{20}$$

where ${\;R}_{ex}$ is the contribution due to chemical exchange between labile protons in biomolecules, and protons in water, and $R_{20}$ is the spin-spin relaxation rate of water without chemical exchange contributions.

Following administration of glucose into the bloodstream, the amount of labile proton exchange between hydroxyl groups (^−^OH) in glucose and water will theoretically increase in proportion with the concentration of glucose, leading to an increase in voxel $R_{1\rho }\;$ via alteration of $R_{ex}$ . The effect of glucose concentration on $R_{20}\;$ is assumed negligible.

Changes in $R_{1\rho }\;$ may occur due to increased/decreased proton exchange with glucose or any other detectable (via exchange) metabolic product of glucose, *i*. We can therefore write the total change in $R_{1\rho }\;$ as the sum of these contributions:

(2) $${\Delta R}_{1\rho }=\sum_{i}r_{i}\Delta C_{i}$$

where $\Delta C_{i}$ is the change in concentration of molecule *i* (*i* includes any detectable molecule, including glucose or metabolic products of glucose) and *r_i_* is the $R_{1\rho }\;$ relaxivity of each of these molecules.

Assuming the population of labile proton *i* is much smaller than the population of water protons, the relaxivities *r*_i_ [(s mM)^−1^] are theoretically dependent on the spin-lock frequency *ω*_1_ (set during the experiment), the resonance frequency gap between molecule *i* and water, *δ*_i_ [s^−1^] (fixed), the number of labile protons per molecule *n*, and the exchange rate between these labile protons and water, *k*_*i*_ [s^−1^] (fixed, but known to vary with tissue pH^46^)

(3) $$r_{i}=(\frac{n}{1.11\cdot 10^{5}})\frac{{k_{i}\delta }_{i}^{2}}{\delta_{i}^{2}+4\pi^{2}\omega_{1}^{2}+k_{i}^{2}}$$

The factor 1.11 × 10^5^ is the concentration of protons in pure water in units of mM (assuming a pure water concentration of 55 M) and ensures the relaxivity has units of [(s mM)^−1^]. For accurate modelling of the relaxivity, the concentration of water protons in the relevant brain structures should be used if known.

In healthy brain tissue, the majority of glucose undergoes glycolysis. In neurons and astrocytes, glucose is phosphorylated by hexokinase into glucose-6-phosphate (G6P), which at rest inhibits hexokinase activity to maintain a constant metabolic rate of glucose consumption, regardless of tissue glucose concentrations.^57^ There may therefore be relatively large fluctuations in G6P following intravenous injection of glucose (in percentage terms), but the basal concentration will remain very low relative to glucose^58^ (<2–3%), and therefore these changes will have only a minor effect on measured ${\Delta R}_{1\rho }$ , even if *r*_*i*_ of 6GP is of a similar magnitude to glucose. At resting state, the concentrations of other downstream metabolic products (e.g. glycogen, glutamate) should remain fairly stable^59^ and independent of changing tissue glucose levels, and thus should also not contribute to measured ${\Delta R}_{1\rho }$ . In tumours, metabolism is reprogrammed to support anabolic growth in nutrient deplete environments. A key component of this transformation is for tumour cells to convert pyruvate to lactate, which then accumulates extracellularly. Fortunately, for glucoCESL studies, the exchange rate of lactate is much lower than the resonance frequency separation between lactate and water^60^ meaning increases in lactate concentration due to increased glucose supply should not contribute significantly to ${\Delta R}_{1\rho }$ (i.e., $k_{i}\ll \delta_{i}^{}$ in equation (3)).

Taken together, we assume that changes in ${\Delta R}_{1\rho }$ following intravenous glucose injection are predominantly driven by changes in glucose concentration only (i.e. effects of changing metabolite concentrations are negligible). We can therefore rewrite equation (2) to include contributions from glucose only, and rearrange to show how measured ${\Delta R}_{1\rho }\;$ can be converted to changes in glucose concentration:

(4) $${\Delta C=\frac{\Delta R_{1\rho }}{r_{g}}}_{}$$

where $r_{g}$ is the $R_{1\rho }$ relaxivity of glucose, and ΔC is the change in tissue glucose concentration. Hence by tracking dynamic changes in $R_{1\rho }\;$ we can infer changes in glucose concentration. Kinetic models can be fit to the concentration time-course data to extract quantitative parameters relating to glucose transport and metabolism.

## Kinetic models

**Figure 1.**
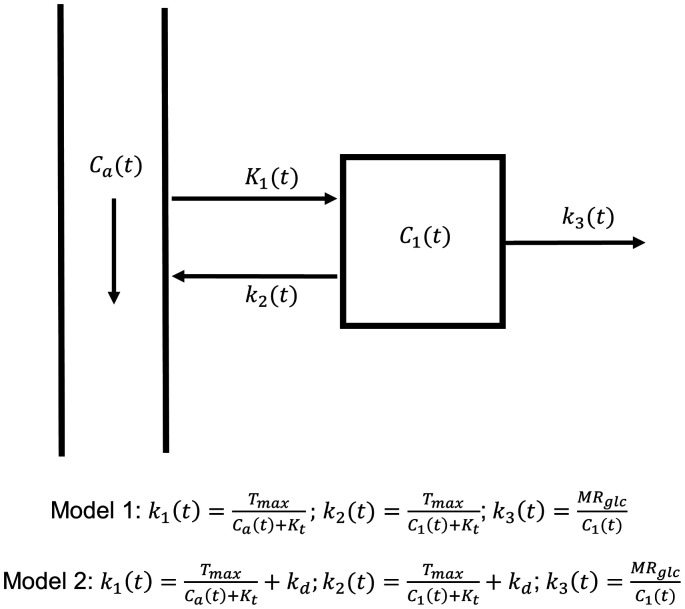
Diagram of the kinetic models. Arterial and tissue glucose have concentrations *C*_a_(t) and *C*_1_(t) respectively. The rate of glucose uptake across an intact blood-brain barrier is modelled with Michaelis-Menten kinetics (model 1), where *k*_1_ is parameterised by the maximal transport capacity *T*_max_, half saturation constant *K*_t_, and is dependent on the arterial glucose concentration *C*_a_(t). In addition to transporter mediated uptake, glucose may also freely diffuse across the BBB into tissue with rate constant *k*_d_ (model 2). We assume that *k*_3_(t) decreases in response to an increase in *C*_1_(t) to maintain a constant metabolic rate of glucose consumption, *MR*_glc_ (i.e., *MR*_glc_ = *k*_3_(t)*C*_1_(t)).

Two kinetic models are proposed to describe changes in tissue glucose concentration (Figure 1). Both models assume the metabolic rate of glucose consumption, *MR*_glc_ = *C*_1_ · *k*_3_, is constant, as would be expected at rest. In the brain, glucose is phosphorylated into glucose-6-phosphate primarily by hexokinase I, and then stored intracellularly before being utilised in the glycolytic or pentose-phosphate pathways.^57^ Hexokinase exhibits unique regulatory properties, in that its rate of activity is potently inhibited by its product.^57,61^ Thus, when glucose-6-phosphate is required by the cell, local glucose-6-phosphate levels become depleted, and the rate of glucose phosphorylation increases to restore glucose-6-phosphate levels (increased *k*_3_). When glucose-6-phosphate levels are high, hexokinase inhibition increases and glucose s.p phosphorylation is reduced (reduced *k*_3_). Thus, when intracellular glucose concentrations are increased (as would occur during an i.v. bolus injection of glucose), the amount of glucose-6-phosphate being produced will momentarily increase, then immediately self-regulate via increased inhibition of hexokinase (reduced *k*_3_).^61^ This mechanism ensures that, at rest and at equilibrium, the metabolic rate of glucose consumption *MR*_glc_ remains constant, even in the presence of increased intracellular glucose levels.

Model 1 assumes the influx and efflux rates of glucose across the blood-brain barrier (BBB), *K*_1_ and *k*_2_, are time-dependent and governed by Michaelis-Menten kinetics.^8^

(5) $$K_{1}(t)=\frac{T_{\max}}{C_{a}\left(t\right)+K_{t}}$$

(6) $$k_{2}(t)=\frac{T_{\max}}{C_{1}\left(t\right)+K_{t}}$$

where *C*_*a*_(t) [mM], and *C*_1_(t) [mM] are the arterial and tissue glucose concentrations respectively, *T*_max_ [µmol/min/mL] is the maximal transport capacity, and *K*_*t*_ [mM] is the half saturation constant of the glucose transporters. Model 2 assumes uptake of glucose is governed by a mixture of Michaelis-Menten kinetics and passive diffusion.^62^ An extra term *k*_d_ [mL/min/mL] is added to the right-hand side of equations (5) and (6) (see Figure 1). The mass transport equation for both models is:

(7) $$\frac{dC_{1}(t)}{dt}=K_{1}(t)C_{a}\left(t\right)-k_{2}(t)C_{1}\left(t\right)-{MR}_{\mathrm{glu}}$$

Both models are defined diagrammatically in Figure 1. The total glucose concentration in a given brain voxel is modelled as a volume weighted average of blood (capillary) and tissue glucose concentrations:

(8) $$C(t)=v_{b}C_{c}\left(t\right)+\;\left(1-v_{b}\right)C_{1}\left(t\right)$$

where *v*_*b*_ [mL mL^−1^] is the fractional blood volume. The capillary glucose concentration is assumed to be equal to the glucose of the feeding artery ( $C_{a}$ = $C_{c}$ ), which is a good assumption in highly perfused tissues such as the brain.^63^

To calculate the change in voxel glucose concentration following intravenous administration of glucose solution, we subtract the modelled baseline glucose concentration:

(9) $$\Delta C\left(t\right)=C\left(t\right)-C(t=0)$$

where C(t=0) can be derived for any compartment model at steady state by assuming $\frac{dC_{1}(t)}{dt}=0$ . Derivations of C(t=0) for model 1 and model 2 are given in Supplementary materials.

## Sensitivity analysis

**Figure 2.**
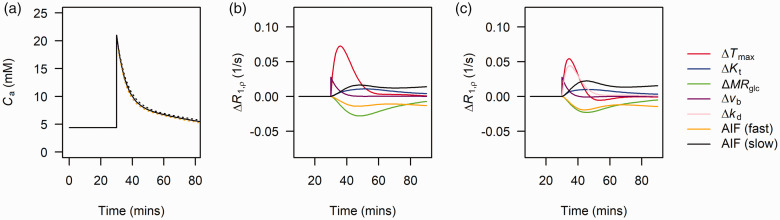
(a) Bi-exponential fit (solid black line) to arterial glucose input functions measured in Sprague-Dawley rats. Dashed yellow and black lines show the effect of simulating a 10% increase and decrease in both λ_1_ and λ_2_ of the Sprague-Dawley population-level curve reflecting faster and slower clearance of glucose from blood respectively. (b) sensitivity plots showing the effects of a 50% increase in each model parameter and increased or decreased blood glucose clearance on Δ*R*_1ρ_ for model 1 and (c) for model 2. While not being varied, parameters were fixed to: *T*_max_ = 3.3 µmol/min/mL, *K*_t_ = 8.1 mM, *MR*_glc_ = 0.3 µmol/min/mL, *k*_d_ = 0.05 mL/min/mL, *v*_b_ = 0.05 ml/mL.

Model sensitivity analysis was performed to determine the relative contribution of each model parameter and the rate of glucose clearance from the blood on $\Delta R_{1\rho }$ . For both kinetic models, a 50% change in each parameter was simulated using equation (9), and the effect of these changes on $\Delta R_{1\rho }$ curves were plotted using equation (4). The initial values of each parameter from which perturbations were made were based on previously published literature data^31,58,64^ and set to *T*_max_ = 3.3 µmol/min/mL, *K*_t_ = 8.1 mM, *MR*_glc_ = 0.3 µmol/min/mL, *k*_d_ =0.05 mL/min/mL, *v*_b_ = 0.05 mL/mL. A population level arterial glucose input function, *C*_a_(t), measured in Sprague-Dawley rats was used (see arterial glucose input function section below for details). Sensitivity of tissue $\Delta R_{1\rho }$ to increased (Figure 2(a); dashed yellow line) or decreased (Figure 2(a), dashed black line) glucose clearance from blood was simulated by creating two additional input functions with *λ*_1_ and *λ*_2_ increased or decreased by 10% from their initial values (see arterial glucose input function section for definitions of *λ*_1_ and *λ*_2_).

## Animals

All animal studies were approved by the Institutional Animal Care and Use Committee at the University of Pittsburgh in accordance with the National Institutes of Health guide for the care and use of laboratory animals. All procedures were conducted in accordance ARRIVE (Animals in Research: Reporting In Vivo Experiments) guidelines (www.nc3rs.org.uk/arrive- guidelines). Animal preparation and MRI experiments have been described previously.^46,50^ Adult male Fischer-344 rats (n = 4, Charles River, Wilmington, MA) weighing between 200-250 g (approx. 12 weeks of age) were anesthetized with 2% isoflurane. A burr hole was drilled into the right hemisphere at stereotaxic coordinates of 0 to 0.5 mm from bregma, and 3 to 3.5 mm from the midline. Inoculation of 100,000 to 300,000 9 L cells (ATCC CRL- 2200, Manassas, VA) in a total volume of 5 µL was performed 4 mm below the surface of the dura. After the induction of tumour cells, rats were imaged 3 to 5 weeks after (approx. age 15–17 weeks), when the tumour reached appropriate volume. Healthy adult male Sprague-Dawley (n = 3, Charles River, Wilmington, MA) rats weighing 335–388 g (approx. 12 weeks of age) were also imaged. An additional n = 3 Fischer-344 rat (254–270 g) were purchased specifically for estimation of a Fischer-344 specific population glucose input function. These animals were not scanned using MRI.

For MRI scans, rats were anesthetized with 2% isoflurane in a mixture of O_2_ and air gases and the right femoral vein catheterised for delivery of glucose. After catheterisation, isoflurane levels were reduced to 1.5%. The dynamic blood pressure, end-tidal CO_2_, and rectal temperature were monitored throughout the duration of the scan. The end- tidal CO_2_ level was kept within 3–4% and the rectal temperature was maintained at 37.5 ± 0.1°C using a feedback-controlled heating pad. MRI data was acquired on a 9.4 T small animal MRI system (Agilent Technologies, Santa Clara, CA, USA) . Radiofrequency transmission was provided by a volume coil (6.4 cm diameter) and received by a surface coil (2.2 cm diameter; coil combination from Nova Medical, MA, USA). Spin-echo EPI CESL images with and without *R*_1ρ_-weighting (i.e., spin-lock pulse duration TSL = 0 and 50 ms) were acquired in an interleaved manner using a spin-lock frequency ω_1_ ∼ 500 Hz. Each set of TSL = 0 ms and TSL = 50 ms images constituted a block, and were used to calculate a single *R*_1ρ_ image as described below.

MRI data for tumour-bearing and healthy rats were acquired in two separate experiments, and some aspects have been previously reported.^46,65^ The time difference between these studies meant there were some differences in the scanning and injection protocols. Table 1 shows the MRI acquisition parameters used for acquisition of tumour-bearing and healthy rat data. All experiments were performed in the non-fasted state. For tumour-bearing rats, glucose was dissolved in distilled water to 30% weight by volume concentration. For healthy rats, glucose was dissolved in distilled water to 20% weight by volume concentration. A glucose bolus (1 g/kg) was injected after 20 minutes (tumour bearing) or 30 minutes (healthy) of baseline data over approximately 30–60 seconds, after which *R*_1ρ_ was measured for 50 minutes. *R*_1ρ_ time-courses were estimated using the following equation:

(10) $$R_{1\rho }=-\frac{1}{0.050\;s}\log\left(\frac{S\left(\mathrm{TSL}=0.050\;s\right)}{S\left(\mathrm{TSL}=0\;s\right)}\right)\;[s^{-1}]$$

**Table 1.** Acquisition parameters for glucoCESL experiments in tumour-bearing and healthy rats.

	Tumour bearing rats (n = 4)	Healthy rats (n = 3)
Field strength	9.4T	9.4T
Readout	Spin-echo EPI	Spin-echo EPI
Number of segment	2	1
TR	2.5s per segment	3s
TE	8.5 ms	30 ms
Field of view	2.56 × 2.56 cm^2^	3.2 × 3.2 cm^2^
Slice thickness	1.5 mm	2 mm
Number of slices	1 or 2 depending on size of tumour	2
Matrix size	96 × 96, interpolated to 128 × 128	64 × 64
Temporal resolution (block size)	30s (2 × TSL = 0, 4 × TSL = 50 ms)	60 s (5 × TSL = 0, 15 × TSL = 50 ms)

## Arterial glucose input functions

Image-based measurement of arterial glucose concentration, *C*_a_(t) (via measurement of Δ*R*_1ρ_(t)), was attempted in each rat by choosing a voxel containing only arterial blood, but results were not reliable. Instead, population level arterial input functions were derived via benchtop arterial blood sampling. For the Sprague-Dawley strain, arterial input functions were obtained the day after MRI scanning under identical experimental conditions. This data has been previously reported in Jin et al.^46^ For the Fischer-344 rats, glucose input functions were not measured at the time of imaging. Three additional Fischer-344 rats were purchased specifically to measure the arterial glucose concentration timecourse, *C*_a_(t).

For each animal, the right femoral artery was catheterized and blood samples acquired every 10 minutes (Sprague-Dawley) or 5 minutes (Fischer-344) following injection of D-glucose. As with MRI experiments, all blood sampling was performed under non-fasting conditions. The same volume, concentration, and injection rate as used during the imaging studies was used to ensure matched experimental conditions. Three blood glucose readings were acquired prior to the glucose injection and averaged to determine the baseline. Before kinetic model fitting, population input functions were smoothed by fitting a bi-exponential model with amplitude and rate parameters *A*_1_, *A*_2_, *λ*_1_, *λ*_2_.

## Error analysis

Systematic error introduced into kinetic model parameters due to use of inaccurate arterial glucose input functions was determined by simulating tissue concentration curves with arterial glucose clearance rates of [A_1_, A_2_, 0.9*λ*_1_ 0.9*λ*_2_] and [A_1_, A_2_, 1.1*λ*_1_ 1.1*λ*_2_] as described above, then fitting back to the simulated curve assuming the original arterial glucose input function (i.e. using [A_1_, A_2_, *λ*_1_ *λ*_2_]). Zero mean gaussian noise with standard deviation, σ, was added to the synthetic curves to give a contrast to noise ratio (ΔC_max_/σ) of 30.

## Kinetic model fitting

Voxelwise estimates of Δ*R*_1ρ_(t) were computed by subtracting the mean pre-injection *R*_1ρ_ and converting to estimates of Δ*C*(t) using equation (4) and *r*_g_ = 0.066 (s mM)^−1^ derived from Figure 1D of Jin et al.^46^ Kinetic models were then fit to Δ*C*(t) time-courses using the ordinary differential equation solver ode45 and lsqcurvefit function in Matlab (Mathworks, version 5 2017a). A delay parameter was included in the fitting routine to adjust for differences in bolus arrival between the population arterial input function and uptake at tissue. Starting parameters and lower and upper limits for fitted parameters were: *T*_max_ = 2 [0, 30] µmol/min/mL, *K*_t_ = 5 [0, 50] mM, *MR*_glc_ = 0.5 [0, 5] µmol/min/mL, *k*_d_ = 0.05 [0, 10] mL/min/mL, *v*_b_ = 0.05 [0, 1].

In tumour-bearing rats, tumour regions of interest (ROIs) and normal tissue ROIs were outlined manually on the TSL = 0 ms image (i.e. the *T*_2_-weighted image) in MRIcron (version 1.0.2). Normal tissue ROIs were drawn on the contralateral side, as to best match the shape and extent of the tumour ROI, but avoiding bright CSF signal. In general these ROIs covered cortical and subcortical gray matter. In healthy rats, normal tissue ROIs were drawn on both sides of the brain and summed together into a single region. These ROIs were drawn to best cover the same regions as the contralateral ROIs drawn for tumour-bearing animals. Prior to comparing tumour and normal tissue parameter values, tests for normality were performed using the Shapiro-Wilks test. The null hypothesis of normality was rejected in less than 5% of data groupings, and thus parametric tests were used throughout.

## ROI-level analyses

The median value of each kinetic parameter was calculated for normal tissue and tumour ROIs. Normal tissue values in healthy rats were combined with contralateral normal tissue values from tumour-bearing rats, and the difference between tumour (n = 4) and normal tissue (n = 7) evaluated using t-tests for partially overlapping samples. To assess heterogeneity in glucose uptake and metabolism within each ROI, the standard deviation of each parameter within tumour and normal tissue ROIs was calculated^66^ and the null hypothesis of no difference tested using t-tests for partially overlapping samples.

The Akaike information criterion (AIC) adjusted for small sample sizes and ΔAIC (ΔAIC = AIC_1_ – AIC_2_) were computed on a voxelwise level to compare the fit quality between models, accounting for differences in the number of fit parameters. To test which model was preferred in tumour and normal tissue, t-tests were performed on ΔAIC to test the null hypothesis that ΔAIC = 0. A t-test for partially overlapping samples was also performed to determine if there was a difference in ΔAIC between tumour and normal tissue. Statistical analysis was done in R (version 4.0.2). Data and analysis scripts used in this paper can be obtained by request to the corresponding author.

## Measurement of the R1_ρ_ relaxivity of lactate

To assess the potential contribution of lactate to Δ*R*_1ρ_ we measured the *R*_1ρ_ relaxivity of lactate in phantom at 9.4T. Lactate phantoms were created with 5 mM, 10 mM, 20 mM and 40 mM of lactate added to 1× phosphate-buffered saline and titrated to pH = 7.0. Phantoms were bundled together and heated to 37°C degree for CESL measurement. The *R*_1ρ_ of each phantom was measured using the following spin-lock pulse parameters: ω_1_ = 500 Hz, varied spin-locking durations (TSL) of 0 to 1 s in 100 ms step. Images were acquired with a spin-echo EPI sequence with the following parameters: one 5-cm slice with FOV = 5 cm × 5 cm, matrix size 64 × 64, TR = 15 s, and TE = 20 ms. The relaxivity of lactate was estimated by plotting *R*_1ρ_ vs lactate concentration and performing linear regression to find the gradient.

## Results

Figure 2(a) shows how the Sprague-Dawley arterial glucose concentration varies with *λ*_1_ and *λ*_2_ set to 0.90 (black dashed) and 1.1 (yellow dashed), simulating slower and faster glucose clearance (for example due to slower and faster insulin responses).

Figure 2(b) and (c) show the results of the Δ*R*_1ρ_ sensitivity analysis for model 1 (saturable transport) and model 2 (mixed saturable transport and free diffusion) respectively. For both models, increases in *T*_max_, *K*_t_, *v*_b_ produced positive Δ*R*_1ρ_, whereas *MR*_glc_ produced negative Δ*R*_1ρ_. A slower glucose clearance from blood produced positive Δ*R*_1ρ_, and faster glucose clearance from blood produced negative Δ*R*_1ρ_. For both models *T*_max_ produced the largest change in Δ*R*_1ρ_ and *K*_t_ the smallest. For model 2, *k*_d_ produced an effect similar in magnitude to *T*_max_ but with a longer tail. For both models, *v*_b_ produced a sharp effect during the first 10 minutes post injection, which rapidly decreased with the concentration of glucose in blood. Changes in the rate of glucose clearance from blood were greater than those due to changes in *K*_t_ (both models) and *MR*_glc_ (model 2 only).

**Figure 3.**
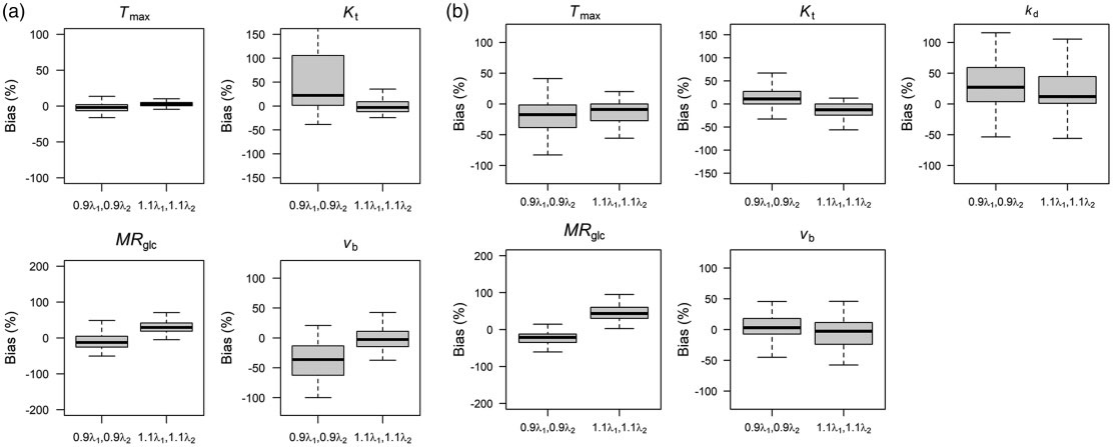
Bias in kinetic parameter estimates due to use of an inaccurate arterial glucose input function for (a) model 1, and (b) model 2 as displayed in Figure 2(a). Data were simulated using Sprague-Dawley input function, then kinetic models refit back to the simulated curves assuming glucose input functions with decreased (0.9λ_1_) and increased (1.1λ_1_) glucose clearance rates.

Figure 3 shows biases in kinetic model parameters due to use of an inaccurate arterial glucose input function. For model 1 (Figure 3(a)), biases were generally less than 40% for all parameters; biases were smallest for *T*_max_ (median less than ±2.6%), and largest for *K*_t_ (−2.8 to +22%), *MR*_glc_ (−13 to +29%), and v_b_ (−36% to +6%). For model 2 (Figure 3(b)), *T*_max_ (−17 to −9%), *K*_t_ (−12 to 11%) and *v*_b_ (−2.8 to +2.9%) were least affected, and *MR*_glc_ (−21 to +45%) and *k*_d_ (+12 to +27%) most affected. Errors in *T*_max_ and *k*_d_ were approximately equal but in opposite directions, possibly reflecting degeneracy in these parameters for describing changes in Δ*R*_1ρ_.

**Figure 4.**
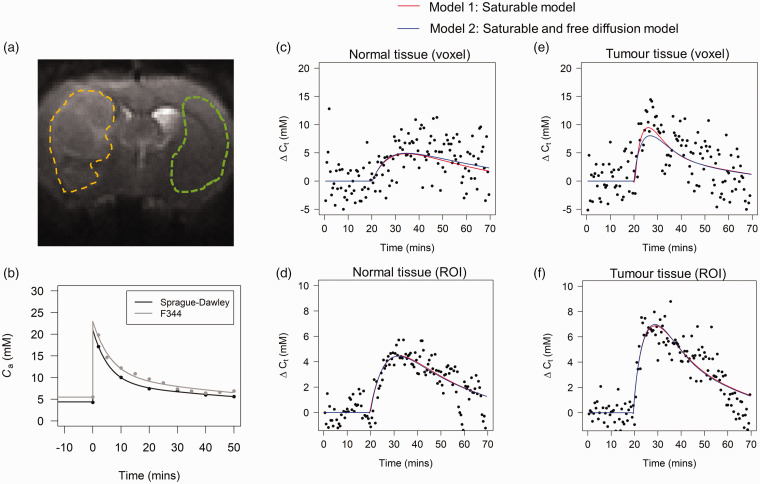
(a) An example CESL image acquired with TSL = 0. These images are T_2_-weighted and provide excellent contrast for definition of tumour (yellow) and contralateral normal tissue (green) ROIs which were drawn manually in MRIcron. (b) Population glucose input functions for the Sprague-Daley and Fisher-344 rats. The curve shapes are similar, but the Fischer-344 curve is ∼1 mM higher. Example voxelwise data and kinetic model fits in normal tissue (c) and tumour (e). Example ROI averaged data and fits in the normal tissue (d) and tumour (f). Both models provide a good fit to normal tissue and tumour data. Model 2 appears to provide a better fit to tumour data at a ROI level.

Figure 4 shows an example of tumour and normal tissue region of interests (ROIs), the population-level input functions and corresponding bi-exponential fit for Sprague Dawley and Fischer-344 rats, and example voxelwise and ROI time-courses and model fits. The estimated parameters from the bi-exponential fits were A_1_ = 12.4 mM, A_2_ = 8.6 mM, *λ*_1_ = 0.18 min^−1^, *λ*_2_ = 0.0086 min^−1^ for Sprague-Dawley, and A_1_ =12.5 mM, A_2_ = 10.4 mM, *λ*_1_ = 0.16 min^−1^, *λ*_2_ =0.0094 min^−1^ for Fischer-344, where A_1_ and A_2_ are the exponential amplitudes, and *λ*_1_ and *λ*_2_ are the decay constants. As expected, ROI-level curves (Figures 4(d) and (f)) had higher signal to noise ratio than voxel-level curves (Figures 4(c) and (e)). In both normal and tumour tissue, model 1 and model 2 provided adequate fit at all timepoints in both voxel and ROI level data. In tumour tissue, model 2 was able to occasionally provide a more accurate description of the peak of the curve (Figure 4E).

**Figure 5.**
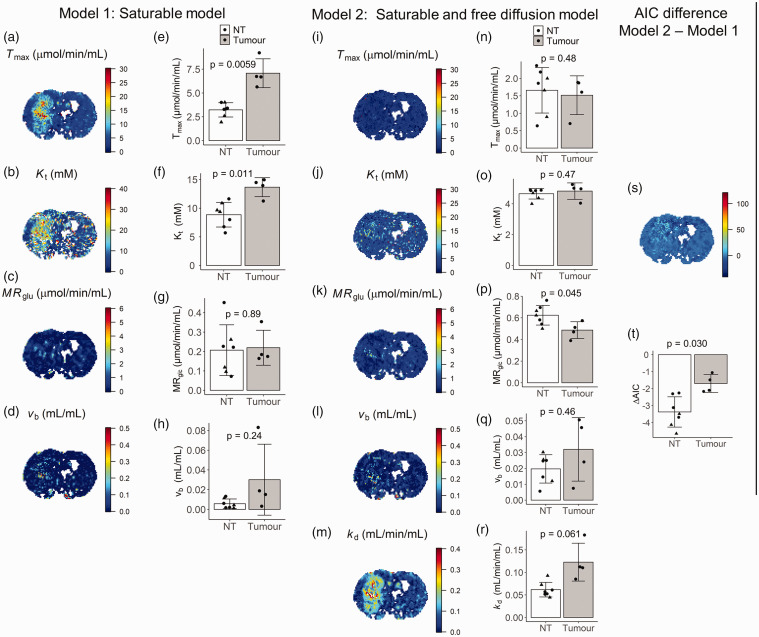
GlucoCESL parameter maps from a tumour bearing rat using model 1 (a–d) and model 2 (i–m), and mean parameter values in normal tissue (healthy – triangle; tumour bearing – circle; n = 7) and tumour (n = 4) for model 1 (e–h) and model 2 (n–r). Far right is the corresponding ΔAIC map (s) and mean ΔAIC in normal tissue and tumour (t). T-tests for partially overlapping samples were used to test the null hypothesis of no difference in parameter values between normal tissue and tumour. Error bars show group standard deviation.

Figure 5 shows spatial maps of each kinetic parameter for a tumour bearing rat, and mean values (±sd) of each kinetic parameter averaged across all rats in normal (n = 7) and tumour tissue (n = 4). P-values show the results of t-tests comparing mean parameters in normal tissue and tumour. Mean values ± sd and p-values are also given for each parameter in Table 2. The difference in AIC between model 1 and model 2 in normal tissue and tumour is also shown as a parameter map (Figure 5(s)), and summarised across all rats as a barplot (Figure 5(t)). Visually, both models produce high fidelity maps of transport and metabolic parameters. For model 1, normal tissue/tumour contrast can be observed in images of *T*_max_ and *K*_t_, whereas little or no contrast is observed for *MR*_glc_ and *v*_b_. This is reflected in the group comparisons. There were significant differences between tumour and normal tissue for *T*_max_ (54% increase; p = 0.0059) and *K*_t_ (40% increase; p = 0.011). On the contrary, there was no significant difference in *MR*_glc_. When evaluating model 2 parameters, there was a borderline significant reduction in *MR*_glc_ in tumour tissue (21% reduction; p = 0.045), and a borderline significant increase in *k*_d_ (49% increase; p = 0.061). Model 2 estimates of *T*_max_, *K*_t_, and *v*_b_ were unchanged between tumour and normal tissue. Averaged across all animals, the difference in AIC between models (AIC model 1–AIC model 2) was significantly less than zero for both normal tissue and tumour tissue (ΔAIC < 0; p = 4.1 × 10^−5^ and p = 0.0076 for normal and tumour tissue respectively).

**Table 2.** Estimates of mean ± sd for kinetic parameters from model 1 and model 2, and results from Partover t-tests between estimates in normal tissue and tumour.

	Model 1 (saturable transport)			Model 2 (saturable and free diffusion)		
	Normal tissue (n = 7)	Tumour (n = 4)	p-value	Normal tissue (n = 7)	Tumour (n = 4)	p-value
*T*_max_ (µmol/min/mL)	3.2 ± 0.6	7.1 ± 2.7	0.0059	1.7 ± 0.67	1.5 ± 0.56	0.48
*K*_t_ (mM)	8.8 ± 2.2	14 ± 1.7	0.011	4.7 ± 0.34	4.8 ± 0.53	0.47
*MR*_glc_ (µmol/min/mL)	0.21 ± 0.13	0.22 ± 0.09	0.89	0.61 ± 0.090	0.48 ± 0.078	0.045
*v*_b_ (mL/mL)	0.0057 ± 0.0048	0.030 ± 0.035	0.24	0.022 ± 0.0087	0.031 ± 0.020	0.46
*k*_d_ (mL/min/mL)				0.061 ± 0.017	0.12 ± 0.042	0.061

Supplementary Figure 1 shows the standard deviation of glucose uptake and metabolic parameters in tumour and normal tissue ROIs. There was no differences in the standard deviation of parameters between normal and tumour tissue except for *v*_b_ from model 1 (p = 0.016; greater sd in tumour tissue), and *k*_d_ from model 2 (p = 0.014; greater sd in tumour tissue).

Supplementary Figure 2 shows how *R*_1ρ_ varies with lactate concentration in phantom. The *R*_1ρ_ relaxivity of lactate was found to be 0.0018 (s mM)^−1^.

## Discussion

We have presented the theory for quantitative kinetic modelling of glucoCESL MRI data, and applied two kinetic models to data from tumour-bearing and healthy rats. Parameter estimates from both models agree well with previously published values.^31^ Mason et al. summarised NMR spectroscopy estimates of *T*_max_, *K*_t_, and *k*_d_ in rat brain from 19 prior studies. In studies that used model 1 (n = 14), estimates of *T*_max_ and *K*_t_ were 3.9 ± 0.5 μmol/min/mL (assuming 0.83 mL water/g tissue) and 8.1 ± 0.6 mM which agree well with our normal tissue estimates of 3.2 ± 0.62 μmol/min/mL and 8.8 ± 2.2 mM. In studies that used model 2 (n = 5), estimates of *T*_max_, *K*_t_, and *k*_d_ were 1.56 ± 0.61 μmol/min/mL, 6.8 ± 0.6 mM, and *k*_d_ = 0.025 ± 0.0074 mL/min/mL, which also agree well with our normal tissue estimates from model 2 of *T*_max_ = 1.7 ± 0.67 μmol/min/mL, *K*_t_ = 4.7 ± 0.34 mM and *k*_d_ = 0.061 ± 0.017 mL/min/mL. While there is greater discrepancy between model 2 parameters and available literature data than for model 1, the direction of change in *T*_max_ and *K*_t_ when using model 2 versus model 1 is consistent (i.e. both are reduced), indicating that our data is also consistent with prior studies.

Model 1 estimates of *MR*_glc_ in normal tissue (0.21 ± 0.13 µmol/min/mL) were also within the range of previously published values. In laboratory rats, the effects of anesthesia on *MR*_glc_ are profound, and must be considered when comparing *MR*_glc_ between studies.^67^ In fasted rats anesthetised with 2% isoflurane, Du et al. measured *MR*_glc_ in a large region covering the cortex and subcortical brain and found values of 0.53 ± 0.20 µmol/min/mL.^28^ Lu et al. used similar anaesthesia and deuterium spectroscopic imaging of deuterated glucose in unfasted rat, and found *MR*_glc_ values in whole rat brain of 0.28 ± 0.13 µmol/min/g.^38^ Shimoji et al. used FDG-PET scanning and halothane anesthesia (1–1.5%) in fasted rat to estimate MR_glc_ in the cortex and found values of 0.24 ± 4.8 µmol/min/g.^68^ Suzuki et al. investigated the effects of different anesthesia on *MR*_glc_ using FDG-PET, including scanning of conscious rat. In conscious rat, much higher values of 0.93 ± 0.18 µmol/min/g were reported, which reduced to 0.54 ± 16 µmol/min/g with isoflurane anethesia.^67^ Similarly, conscious rat values for *MR*_glc_ obtained by Hawkin’s et al. using the autoradiographic technique were of the order of 1.1–1.2 µmol/min/g.^64^ Thus, our values for *MR*_glc_ certainly lie within the range of previously published values for *MR*_glc_ in anesthetised rats.

Model 2 estimates of *MR*_glc_ (0.61 ± 0.009 µmol/min/mL) were approximately 3 times higher than *MR*_glc_ estimates made using model 1 (0.21 ± 0.13 µmol/min/mL). It is possible that the introduction of the additional *k*_d_ parameter could lead to more accurate modelling of glucose influx/efflux, leading to more accurate estimation of *MR*_glc_. We assessed other studies using model 2 to determine if *MR*_glc_ was affected. Pardridge et al. used pentabarbitol anesthetisia and found *MR*_glc_ values in cortex of 0.28 µmol/min/mL.^69^ Crane et al. also used pentabarbitol anesthesia but also studied conscious rat and found values of *MR*_glc_ of 0.27 μmol/min/g^21^ and 1.02 μmol/min/g respectively. Cremer studied conscious rat only and found values of 1.24 μmol/min/mL.^70^ While data is not available on isoflurane-anesthetised rats, all these values are not substantially different from literature values using model 1 indicating that model 2 estimates of *MR*_glc_ should not be affected substantially by modelling of free diffusion, particularly in tissues with intact BBB. It is therefore important that further work is done to validate model 2 estimates of *MR*_glc_ obtained using glucoCESL MRI.

The maximal transport capacity (*T*_max_) and the half saturation constant (*K*_t_) from model 1 were significantly higher in tumour than normal tissue, indicating increased uptake of glucose. Higher *T*_max_ may reflect an increased density of glucose transporters on the vasculature and tumour cell membranes. However, in the context of increased *K*_t_, the interpretation must be carefully considered. *K*_t_ will trade-off saturable and non-saturable kinetics, and as *K*_t_ increases, *K*_1_ becomes less dependent on *C*_a_, and begins to mimic the behaviour of the free diffusion constant *k*_d_. Thus, increased *T*_max_ and *K*_t_ together may reflect the presence of free diffusion and blood-brain barrier breakdown. This interpretation is supported by model 2 parameters, where differences in *T*_max_ and *K*_t_ between tumour and normal tissue do not exist or are much smaller, and variability in uptake of glucose is described mainly by *k*_d_ (Figure 5(a), (b), (i), (j) and (m)). We did not find any difference in metabolic rate of glucose consumption between tumours and normal tissue for model 1, which is consistent with the clinical observation that uptake of FDG in many brain tumours is often no higher than surrounding normal tissue.^16^ On the contrary, a small but significant reduction in model 2 *MR*_glc_ was observed in tumour tissue. However, given the uncertainty associated with model 2 estimates of *MR*_glc_ as described previously, this result should be treated with caution until verified by further studies. Model 1 and model 2 predicted no difference in blood volume between normal tissue and tumour. Model 1 estimates of blood volume were much lower than known values (v_b_ < 0.005 mL/mL and v_b_ < 0.01 mL/mL for normal tissue and tumour respectively). Estimates for model 2 were more reasonable but still low ∼ 0.02–0.03 mL/mL. The underestimation could be a result of the shorter *T*_2_ of the blood compartment relative to tissue at 9.4 T^71^ which might cause glucose in the blood compartment to be less visible than glucose in tissue.

Estimates of the Akaike information criterion (AIC) in normal and tumour tissue showed that model 1 had significantly lower AIC than model 2 (ΔAIC < 0; p = 4.1 x10^−5^ and p = 0.0076 for normal and tumour tissue respectively), indicating that the additional free diffusion parameter of model 2 did not describe any additional variability above that described by model 1. This does not mean that model 2 is less accurate or did not fit the data as well as model 1, but that within the conditions of our experiment (i.e. temporal resolution, SNR), the additional parameter did not provide additional predictive power over parameters already present in model 1. It is clear from our results that the normal tissue values of transport and metabolic parameters from model 1 are more consistent with literature values. It was also shown in previous work that the same tumour-bearing rats as scanned in this study exhibit leakage of DCE-MRI contrast agents (see Figure 3C of Jin et al.^65^) indicative of BBB breakdown. This suggests model 2 may more appropriately ascribe changes in glucose uptake in tumour tissue to free diffusion rather than changes to saturable transport mechanisms. Further validation work is needed to confirm these points.

We have assumed that contributions from metabolic products of glucose to Δ*R*_1ρ_ were negligible compared to the effects of glucose itself. It is shown in previous work that changes in concentrations of glucose-6-phosphate^58^ and neurotransmitters such as glutamate^59^ are very small, and should not affect Δ*R*_1ρ_. Since tumours can produce high levels of lactate, we performed an additional phantom study to demonstrate that rising lactate levels will also not appreciably affect Δ*R*_1ρ_. The *R*_1ρ_ relaxivity of lactate was found to be 0.0018 (s mM)^−1^, 37 times smaller than glucose. Using the reported values of the chemical shift and exchange of lactate from recent literature,^60^ predicted *R*_1ρ_ relaxivity is even smaller than our measured value (∼0.0005 (s mM)^−1^, 134 times smaller than glucose). It is not yet clear what effects lactate will have on CEST detection, which is more sensitive to slower exchange rates than CESL. Changes to Δ*R*_1ρ_ from osmolality effects and differences in tissue pH were also ignored, which may have a larger effect on Δ*R*_1ρ._^50^ These factors, and the impact of a wider range of metabolites, should be investigated in future work to further refine the modelling approach.

The study has the following limitations. We have used a population-based arterial glucose input function instead of using individually sampled, or image-derived arterial input functions. We attempted to obtain image-derived AIFs from the carotid artery, but it was not possible to obtain reliable results. This is presumably due to inflow of fresh unsaturated blood into the imaging slice (i.e. blood that has not undergone spin-locking) during the time between spin-locking and the SE-EPI readout. It is possible the shape of the population level curves may have deviated from the true (individual) input function of the rats investigated in this study. Our simulations showed that deviations between assumed and true arterial glucose concentrations can have appreciable effects on Δ*R*_1ρ_, particularly at later time points (the peak effect was observed ∼20 minutes post glucose injection), which then propagate through to biases in estimates of all kinetic parameters, but predominantly affected *K*_t_, *MR*_glc_, and *v*_b_ for model 1 and *MR*_glc_ and *k*_d_ for model 2. These results highlight the importance of accurate input function characterisation when using such models, particularly for inter-subject or group-level comparisons. Accurate knowledge of input functions may not be so important for intra-subject comparisons (e.g. tumour vs normal tissue) as it could be expected the glucose input to different brain regions will be similar. Despite the potential for large biases, the excellent correspondence between our kinetic parameter estimates (particularly for model 1) and literature values suggest that the input functions used in this work are an accurate representation of the true arterial glucose time-courses. Future work should investigate the use of CESL and CEST approaches for measuring an image-derived input functions, and/or further validate the accuracy of the population based input function approach for group level comparisons. It is possible that adjustment (e.g. scaling) of population level input functions using pre- and post-scan venous blood glucose measurements (e.g. using a simple blood glucose monitor) may be adequate to account for inter-individual or group variability in blood glucose concentration time-courses. There were some small differences in the acquisition parameters and injection protocols between tumour bearing and healthy rats. For example, the weight by volume concentration, and hence the administered volumes of glucose solution were different. It is possible these effects led to small differences in osmolality of blood. However, given the estimated kinetic parameter values for normal tissue in healthy and tumour bearing rats were not appreciably different, we are confident these effects were small. For future glucose-enhanced MRI studies, including translational work in humans, a standardised set of optimal acquisition parameters are needed.

To conclude, we have for the first time demonstrated the feasibility of applying kinetic models to glucoCESL MRI data and show the potential of the approach to probe glucose transport and metabolism *in-vivo* at high spatial resolution.

## Supplemental Material

sj-pdf-1-jcb-10.1177_0271678X221108841 - Supplemental material for Quantitative kinetic modelling and mapping of cerebral glucose transport and metabolism using glucoCESL MRI Click here for additional data file. Supplemental material, sj-pdf-1-jcb-10.1177_0271678X221108841 for Quantitative kinetic modelling and mapping of cerebral glucose transport and metabolism using glucoCESL MRI by Ben R Dickie, Tao Jin, Ping Wang, Rainer Hinz, William Harris, Hervé Boutin, Geoff JM Parker, Laura M Parkes and Julian C Matthews in Journal of Cerebral Blood Flow & Metabolism
