# Supplementary material for: Quantitative kinetic modelling and mapping of cerebral glucose transport and metabolism using glucoCESL MRI
Source: J Cereb Blood Flow Metab. 2022 Jun 23;42(11):2066–79. doi: 10.1177/0271678X221108841 (PMC9580170; doi:10.1177/0271678X221108841)
Supplement: sj-pdf-1-jcb-10.1177_0271678X221108841 - Supplemental material for Quantitative kinetic modelling and mapping of cerebral glucose transport and metabolism using glucoCESL MRI [file sj-pdf-1-jcb-10.1177_0271678X221108841.pdf]

SUPPLEMENTARY INFORMATION

Derivation of baseline glucose concentration in tissue, $C_1(0)$

The baseline (pre-injection) tissue concentration in tissue, $C_1(0)$, can be calculated by fixing the rate of change of tissue glucose concentration to 0 in Eqn 7 and solving for $C_1(0)$.

For model 1 assuming saturable transport and free diffusion:

$$\frac{dC_1(0)}{dt} = \frac{T_{max}}{K_t + C_a(0)} C_a(0) - \frac{T_{max}}{K_t + C_1(0)} C_1(0) - MR_{glc} = 0$$

$$C_1(0) = K_t \frac{T_{max} C_a(0) - \ MR_{glc}(C_a(0) + K_t)}{MR_{glc}(C_a(0) + K_t) + \ K_t T_{max}}$$

where $C_a(0)$ [mM] is the baseline (pre-injection) glucose concentrations respectively, T_{max} [µmol/min/mL] is the maximal transport capacity, K_t [mM] is the half saturation constant of the glucose transporters, and MR_{glc} is the cerebral metabolic rate of glucose consumption.

For model 2 assuming saturable transport and free diffusion:

$$\frac{dC_1(0)}{dt} = (\frac{T_{max}}{K_t + C_a(0)} + k_d) C_a(0) - (\frac{T_{max}}{K_t + C_1(0)} + k_d) C_1(0) - MR_{glc} = 0$$

$$((\frac{T_{max}}{K_t + C_a(0)} C_a(0) + k_d C_a(0) - MR_{glc}) \ (K_t + C_1(0)) - (T_{max} + k_d\big(K_t + C_1(0)\big) C_1(0) = 0$$

$$-k_d C_1(0)^2 + \left(\frac{T_{max}}{K_t + C_a(0)} C_a(0) + k_d C_a(0) - MR_{glc} - T_{max} - k_d K_t\right) C_1(0)$$

$$+ \left(\frac{T_{max} K_t}{K_t + C_a(0)} C_a(0) + k_d K_t C_a(0) - MR_{glc} K_t\right) = 0$$

$$a = -k_d$$

$$b = \frac{T_{max}}{K_t + C_a(0)} C_a(0) + k_d C_a(0) - MR_{glc} - T_{max} - k_d K_t$$

$$c = \frac{T_{max} K_t}{K_t + C_a(0)} C_a(0) + k_d K_t C_a(0) - MR_{glc} K_t$$

$$C_1(0) = \frac{-b - \sqrt{b^2 - 4ac}}{2a}$$

Where k_d is the free diffusion constant [mL/min/mL].

Supplementary Figures

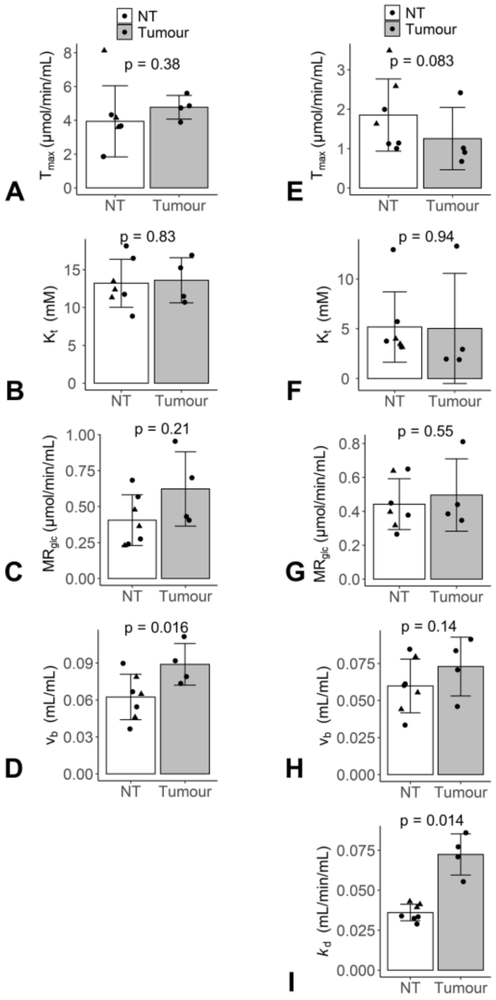

Supplementary Figure 1. Standard deviation of parameter values as a marker of heterogeneity in normal tissue (NT; healthy – triangle; tumour bearing – circle; n = 7) and tumour (n = 4 - circle) for model 1 (A-D) and model 2 (E-I). T-tests for partially overlapping samples were used to test the null hypothesis of no difference in the heterogeneity of parameter values between normal tissue and tumour. Error bars show group standard deviation.

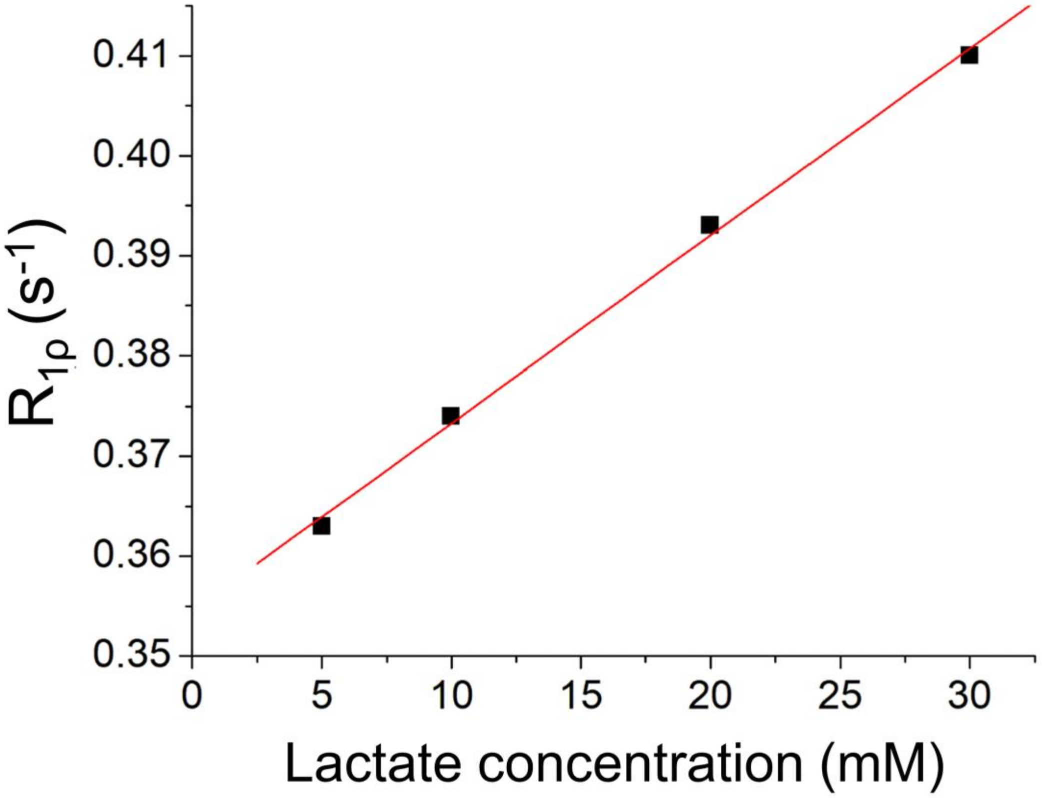

Supplementary Figure 2. Measurement of the $R_{1\rho}$ relaxivity of lactate. $R_{1\rho}$ is shown to be linearly proportional to lactate concentration (at pH 7.0); the slope of the linear fit is 0.0018 (s mM)$^{-1}$.
